# Analysis of hospitalization costs for a TCM-advantaged disease under DRG payment reform: a case study of Xiang Bi

**DOI:** 10.3389/fpubh.2026.1902363

**Published:** 2026-10-21

**Authors:** Xihao Zhang, Ke Ding, Yue Lv, Wei Zhang

**Affiliations:** 1 School of Health Management, Gansu University of Chinese Medicine, Lanzhou, China; 2 First Affiliated Hospital of Zhejiang Chinese Medical University (Zhejiang Provincial Hospital of Chinese Medicine), Medical Insurance and Price Office, Zhejiang, China

**Keywords:** DRG, DRG payment reform, generalized linear model, hospitalization costs, TCM-advantaged diseases

## Abstract

**Objective:**

This study aims to analyze the composition, temporal trends, and influencing factors of hospitalization costs for “Xiang Bi” (cervical spondylosis), a TCM-advantaged disease category, against the background of ongoing DRG payment reform and the deepening of policies supporting TCM, so as to provide empirical evidence for improving medical insurance payment mechanisms that align with the characteristics of TCM.

**Methods:**

A total of 1,046 inpatients discharged with a diagnosis of Xiangbi between January 1, 2023, and December 31, 2025, from a tertiary TCM hospital in Z province were selected. Data were collected from the first pages of medical records and detailed medical insurance settlement records. Descriptive analysis, Mann–Whitney U test, Kruskal–Wallis H test, and generalized linear models were used to statistically analyze the composition, annual changes, and influencing factors of hospitalization costs.

**Results:**

Patients hospitalized with Xiangbi were predominantly young and middle-aged women, and the application rate of TCM characteristic techniques reached 94.0%. From 2023 to 2025, the average total hospitalization cost per visit decreased from 10,610.66 yuan to 7,273.65 yuan, showing a continuous downward trend during the three-year study period. The proportion of TCM treatment costs rose from 22.22 to 32.46%, becoming the dominant cost component, while the proportions of diagnostic tests, Western medicine, and consumables decreased to 18.79, 6.56, and 13.58%, respectively. The generalized linear model revealed that length of stay (Wald χ^2^ = 117.860, *p* < 0.001), year (Wald χ^2^ = 10.859, *p* = 0.004), age group (Wald χ^2^ = 23.799, *p* = 0.001), application of TCM characteristic techniques (Wald χ^2^ = 7.240, *p* = 0.007), use of hospital-prepared TCM preparations (Wald χ^2^ = 9.302, *p* = 0.002), and use of TCM diagnostic and therapeutic equipment (Wald χ^2^ = 7.539, *p* = 0.006) were independent influencing factors of hospitalization costs.

**Conclusion:**

During the implementation of the DRG payment reform, a year-by-year decreasing trend in the total hospitalization costs for Xiangbi was observed, with the cost structure showing an optimized trend driven by TCM techniques and a compressed proportion of drugs and consumables. TCM characteristic techniques exhibited potential advantages in cost containment; however, factors such as length of stay and age still significantly affected the cost level. It is recommended that length of stay be adopted as a core control target, incentive mechanisms reflecting the labor value of TCM characteristic techniques be refined, differentiated tiered payment strategies be established, and multicenter, long-term cost evaluation studies be advanced. Nevertheless, because this study lacked control data from before the reform, the changes observed cannot be directly attributed to the DRG reform. Future research should incorporate quasi-experimental designs such as interrupted time series or difference-in-differences analyses for verification. This study is intended to provide a descriptive basis and generate hypotheses for future causal inference research, rather than to serve as a substitute for rigorous policy-effect evaluation.

## 1 Introduction

With the deepening reform of China’s healthcare security system, the reform of medical insurance payment methods has become a core issue. Over the years, China’s Traditional Chinese Medicine (TCM) policies have been continuously deepened, systematically enhancing TCM service capabilities, standardizing clinical practices, and innovating payment models, thus laying a solid institutional foundation for the development and application of core TCM-advantaged diseases. In 2019, the Central Committee of the Communist Party of China and the State Council issued the Opinions on Promoting the Inheritance, Innovation, and Development of Traditional Chinese Medicine, which made a pivotal deployment by explicitly stating the need to “select, in batches, diseases with obvious TCM advantages, clear treatment pathways, and definite costs for case-based payment,” directly linking TCM-advantaged diseases with medical insurance payment reform at the national level for the first time (1). In 2023, the General Office of the State Council, in the Implementation Plan for Major Projects to Revitalize and Develop Traditional Chinese Medicine, once again specified the organized screening of 50 TCM-advantaged diseases (2). In 2024, the Key Tasks for Deepening the Reform of the Medical and Health System in 2024 issued by the General Office of the State Council listed “piloting payment for TCM-advantaged diseases” as an annual priority (3). In 2025, the National Healthcare Security Administration and the National Administration of Traditional Chinese Medicine jointly issued the Notice on Launching the Pilot Work of Case-Based Payment for TCM-Advantaged Diseases and the Recommended Catalogue of TCM-Advantaged Diseases Suitable for Case-Based Payment, specifying 56 TCM-advantaged diseases, including “Xiangbi (cervical spondylosis)”, and corresponding payment principles, signifying the transition of case-based payment for TCM-advantaged diseases from policy conception into a substantive, catalogued pilot promotion stage (4, 5). Although research on the impact of DRG-based payment on hospital operations has been gradually increasing (6), most studies focus on Western medicine diseases. In contrast, China’s TCM policy system has formed a clear evolutionary path from “standardizing clinical pathways” to “building advantageous specialties” and then to “innovating medical insurance payment” (7), providing a complete policy basis and practical context for the in-depth analysis of hospitalization costs for specific TCM-advantaged diseases such as “Xiangbi” against the backdrop of the DRG payment reform (8). Therefore, this study integrates national and local policy orientations regarding DRG reform and TCM-advantaged diseases, using “Xiangbi” as an example to analyze its hospitalization cost characteristics and medical insurance payment optimization pathways during the DRG payment reform, aiming to provide empirical evidence for improving medical insurance payment policies that align with the features of TCM.

## 2 Data and methods

### 2.1 Data source

The data for this study were obtained from the hospital information system (HIS) and the medical insurance settlement system of a tertiary Grade A Traditional Chinese Medicine (TCM) hospital in Z Province. Z Province is located in the East China region and is an economically developed province. It initiated the DRG payment reform in 2020, and by the end of 2023, full DRG payment coverage had been achieved for all eligible inpatient medical institutions across the province. The hospital in this study is a tertiary Grade A TCM hospital in Z Province, serving as the flagship institution of the province’s TCM service system, with its comprehensive strength ranking among the top TCM hospitals in the province. The study period (January 1, 2023 to December 31, 2025) falls entirely within the post-full-implementation phase of the DRG reform in Z Province, as full coverage was achieved by the end of 2023.

Data on the front pages of medical records and detailed medical insurance settlement data were collected for all inpatients discharged from the Department of Orthopedics and Traumatology between January 1, 2023 and December 31, 2025, whose TCM diagnosis was neck impediment and whose Western medicine diagnosis was any of the following: sympathetic cervical spondylosis, cervical spondylotic radiculopathy, vertebral artery type cervical spondylosis, mixed cervical spondylosis, cervical disc herniation, cervical disc herniation with radiculopathy, cervical spondylosis, cervical degenerative disease, cervical disc herniation with myelopathy, cervical and thoracic intervertebral disc disorders with radiculopathy, and cervical disc disorders. The diagnostic codes of the selected discharge diagnoses were all chosen in accordance with the “Notice on the Issuance of the Recommended Catalogue of TCM Dominant Diseases Suitable for Payment by Disease” issued by the Comprehensive Department of the National Administration of Traditional Chinese Medicine and the General Office of the National Healthcare Security Administration.

The data extraction and database lock date was January 15, 2026. Data cleaning was completed on March 27, 2026, and statistical analysis was performed on April 20, 2026. All data had been collected and locked prior to the analysis, and there were no instances of incomplete data.

### 2.2 Inclusion and exclusion criteria

#### 2.2.1 Inclusion criteria

(1) The primary diagnosis was *Xiangbi* (cervical spondylosis) as defined in the treatment protocol of the National Administration of Traditional Chinese Medicine, or a Western medicine diagnosis of sympathetic cervical spondylosis (M47.202), cervical spondylotic radiculopathy (M47.201), vertebral artery type cervical spondylosis (M47.001 + G99.2\*), mixed cervical spondylosis (M47.8), cervical disc herniation (M50.2), cervical disc herniation with radiculopathy (M50.101+), cervical spondylosis (M47.801), cervical degenerative disease (M48.901), cervical disc disorder with myelopathy (M50.0), cervical and thoracic intervertebral disc disorder with radiculopathy (M50.1), or cervical disc disorder, unspecified (M50.9);
(2) The medical insurance type was the Basic Medical Insurance for Urban Employees of Z Province, the Basic Medical Insurance for Urban and Rural Residents, or the New Rural Cooperative Medical Scheme;
(3) The treatment modality included traditional Chinese medicine (TCM) or integrated traditional Chinese and Western medicine treatment.

#### 2.2.2 Exclusion criteria

(1) Cases with a length of hospital stay <1 day or >60 days;
(2) Cases with missing key information on the front page of the medical record or with obvious logical errors;
(3) Cases without a definite diagnosis;
(4) Cases with missing data on key variables (such as costs, diagnosis, and length of stay) or with obvious logical contradictions (e.g., negative costs, discharge date earlier than admission date).

According to the inclusion and exclusion criteria, a total of 1,046 cases were finally included in this study.

### 2.3 Research methods

This study collected the front pages of medical records and detailed expense data of inpatients with Xiangbi (cervical spondylosis) between January 1, 2023 and December 31, 2025, through the hospital information system and the medical insurance settlement system. Data sorting and cleaning were performed using Microsoft Excel 2020, and statistical analyses were conducted with SPSS 27.0. Therefore, all cases in this study were treated under the DRG payment system.

Statistical analysis methods: Descriptive analyses were performed to summarize the basic characteristics of patients and the composition of costs. Categorical variables were presented as frequencies and proportions (%), while non-normally distributed continuous variables were described using medians (M) and interquartile ranges (IQR). Because total hospitalization cost exhibited a markedly right-skewed distribution, the Mann–Whitney U test was used for two-group comparisons and the Kruskal–Wallis H test for multiple-group comparisons in univariate analysis, with a significance level of α = 0.05.

Multivariable model specification: To identify independent influencing factors of hospitalization cost and adjust for confounders, a generalized linear model (GLM) was fitted with total hospitalization cost as the dependent variable. Given the large variance, non-negativity, and skewed nature of the cost data, a Gamma distribution with a log link function was selected. Variables with *p* < 0.05 in univariate analysis, along with factors considered clinically meaningful, were simultaneously entered into the model.

Model evaluation metrics: The overall significance of the model was assessed using the omnibus likelihood ratio test, and the effect of each independent variable was tested using the Type III Wald chi-square test. Goodness of fit was evaluated by the ratio of deviance to degrees of freedom and the ratio of Pearson chi-square to degrees of freedom. Values of both ratios less than 1 indicated an adequate model fit without obvious overdispersion.

To assess potential multicollinearity among the independent variables, variance inflation factor (VIF) diagnostics were performed prior to final model fitting. A generalized VIF (GVIF) approach was applied given the presence of categorical variables with multiple levels. All VIF values were below the conventional threshold of 5, indicating that multicollinearity was not a substantial concern and did not compromise the stability of the parameter estimates.

The generalized linear model included 13 independent variables (with categorical variables coded as dummy variables, totaling 20 degrees of freedom). For models with continuous outcomes, the effective sample size can be evaluated using the events per variable (EPV) criterion adapted for continuous data, where the “event” is defined as each observation. The EPV for this analysis was 1046/20 = 52.3, which substantially exceeds the commonly recommended minimum of 10 EPV, indicating that the sample size was sufficient to support stable estimation of the multivariable model.

## 3 Research contents

### 3.1 Basic characteristics of inpatients

A total of 1,046 inpatients with Xiangbi (cervical spondylosis) were included in this study. In terms of sex distribution, there were 440 males (42.1%) and 606 females (57.9%). The patients were predominantly young and middle-aged adults aged 31–60 years (accounting for 71.8%), with the 41–50 age group having the largest number of patients (275, 26.3%). Regarding marital status, 900 patients (86.0%) were married. The main payment method for medical expenses was the Basic Medical Insurance for Urban Employees (850 patients, 81.3%). The length of hospital stay was concentrated within 1–13 days (970 patients, 92.7%), and 91.3% (955 patients) had only one hospitalization episode. The distribution by year of admission showed an increasing trend in the number of cases year by year (212 in 2023, 312 in 2024, and 522 in 2025). The admission route was predominantly through outpatient clinics (1,033 patients, 98.8%). The vast majority of treatment categories involved integrated traditional Chinese and Western medicine (1,030 patients, 98.5%). The most common mode of discharge was discharge upon medical advice (990 patients, 94.6%). Patients who received characteristic traditional Chinese medicine (TCM) techniques accounted for 94.0% (983), those who used hospital-based TCM preparations accounted for 82.5% (863), and those who used TCM diagnostic and therapeutic equipment accounted for 71.5% (748), indicating that TCM technologies were commonly applied among inpatients with Xiangbi (see Table 1).

**Table 1:** Basic information of hospitalized patients.

Indicator		Number of cases	%
Gender	Male	440	42.1
	Female	606	57.9
Age	≤30	110	10.5
	31–40	265	25.3
	41–50	275	26.3
	51–60	211	20.2
	61–70	120	11.5
	71–80	62	5.9
	≥81	3	0.3
Marital status	No spouse	146	14.0
	With spouse	900	86.0
Medical payment method	Urban resident basic medical insurance	76	7.3
	Urban employee basic medical insurance	850	81.3
	New rural cooperative medical system	120	11.5
Length of stay (days)	1 ~ 13	970	92.7
	≥14	76	7.3
Number of hospitalizations	1time	955	91.3
	≥2times	91	8.7
Year	2023	212	20.3
	2024	312	29.8
	2025	522	49.9
Admission route	Emergency	13	1.2
	Outpatient	1,033	98.8
Treatment category	Traditional Chinese Medicine	16	1.5
	Integrated TCM and Western medicine	1,030	98.5
Discharge method	Discharge by doctor’s advice	990	94.6
	Transfer by doctor’s advice	56	5.4
Application of TCM characteristic techniques	Yes	983	94.0
	No	63	6.0
TCM hospital preparations	Yes	863	82.5
	No	183	17.5
TCM diagnostic and treatment equipment	Yes	748	71.5
	No	298	28.5

### 3.2 Cost structure of inpatients

The total hospitalization costs and cost category composition for patients with Xiangbi are shown in Table 2. From 2023 to 2025, the average total hospitalization cost per visit decreased from 10,610.66 yuan to 7273.65 yuan, demonstrating a continuous downward trend. Regarding cost composition, the proportion of TCM treatment-related costs increased from 22.22 to 32.46%, with an average annual increase of approximately 5 percentage points, making it the largest cost category since 2024. The proportion of diagnostic examination costs decreased from 20.20 to 18.79%. The proportion of consumables costs experienced the largest decline, dropping from 20.48 to 13.58%. The proportion of Western medicine costs decreased from 9.46 to 6.56%. The proportions of comprehensive medical service costs, Chinese herbal medicine costs, rehabilitation costs, and other costs remained relatively low, with minimal fluctuations across the years. These results indicate that the cost structure exhibited an optimization trend, characterized by a strengthened driving role of TCM treatment-related costs and a continuously compressed share of pharmaceutical and consumables costs.

**Table 2:** Structure of patients’ hospitalization costs.

Cost category	2023		2024		2025		Total	
Diagnostic and examination	2143.30	20.20%	1491.01	18.10%	1366.75	18.79%	1561.20	18.95%
Comprehensive medical services	588.12	5.54%	510.54	6.20%	423.21	5.82%	482.68	5.86%
Western medicine	1003.41	9.46%	624.73	7.58%	477.19	6.56%	627.85	7.62%
Traditional Chinese medicine	493.66	4.65%	468.80	5.69%	417.66	5.74%	448.32	5.44%
Treatment (excluding TCM treatment)	1357.16	12.79%	952.19	11.56%	904.43	12.43%	1010.43	12.27%
TCM treatment	2357.81	22.22%	2624.80	31.86%	2360.80	32.46%	2438.94	29.61%
Consumables	2173.07	20.48%	1150.95	13.97%	987.40	13.58%	1276.49	15.50%
Rehabilitation	447.10	4.21%	386.13	4.69%	308.65	4.24%	359.82	4.37%
Other	47.03	0.44%	29.74	0.36%	27.57	0.38%	32.16	0.39%
Total cost	10610.66	100.00%	8238.90	100.00%	7273.65	100.00%	8237.90	100.00%

### 3.3 Univariate analysis of hospitalization costs for patients with Xiangbi

A univariate analysis was conducted with total hospitalization cost as the dependent variable. The results showed that the differences in hospitalization costs among groups stratified by age, marital status, length of stay, year, treatment category, application of TCM characteristic techniques, and use of hospital-prepared Chinese herbal preparations were statistically significant (all *p* < 0.05). Specifically, hospitalization costs tended to increase with age (*p* < 0.001), with the highest average cost observed in the 71–80 age group (10599.52 yuan). Patients with a length of stay ≥14 days had a substantially higher average cost (20246.30 yuan) than those with a stay of 1–13 days (7297.03 yuan, *p* < 0.001). Significant differences in costs were also observed across years (*p* < 0.001), with the highest average cost in 2023 (10610.66 yuan) and the lowest in 2025 (7273.65 yuan). Patients who received TCM characteristic techniques had a lower average cost than those who did not (*p* < 0.001), whereas patients who used hospital-prepared Chinese herbal preparations had a higher average cost than non-users (*p* < 0.001). No statistically significant differences in hospitalization costs were found among groups defined by sex, number of hospitalizations, medical payment method, admission route, discharge method, or application of TCM diagnostic and therapeutic equipment (*p* > 0.05) (see Table 3 for details).

**Table 3:** Univariate analysis of patients’ hospitalization costs.

Independent variable	Group	Mean	Median	IQR	*P*
Gender	Male	9128.60	6350.40	5438.89	0.271
	Female	7591.18	6388.55	4425.16	
Age	≤30	5282.29	4581.08	3478.65	<0.001
	31–40	7556.97	5940.89	4946.56	
	41–50	8094.59	6515.61	4959.97	
	51–60	8771.50	6802.79	4389.41	
	61–70	10584.60	7593.46	6418.15	
	71–80	10599.52	7341.58	6489.18	
	≥81	9689.95	7399.73	.	
Marital status	No spouse	8478.78	6582.74	4827.66	<0.001
	With spouse	6753.01	5112.81	4541.15	
Length of stay (days)	1 ~ 13	7297.03	6028.69	4160.58	<0.001
	≥14	20246.30	15842.87	12830.36	
Number of hospitalizations	1time	8198.29	6382.78	4815.11	0.631
	≥2times	8653.51	6785.92	5172.65	
Medical payment method	Urban resident basic medical insurance	10108.30	7004.49	8640.44	0.071
	Urban employee basic medical insurance	7798.29	6252.23	4506.95	
	New rural cooperative medical system	10167.21	6905.88	6886.91	
Year	2023	10610.66	7448.79	7129.21	<0.001
	2024	8238.90	6202.21	4916.98	
	2025	7273.65	5978.74	4481.83	
Admission route	Emergency	8988.51	5773.02	5180.14	0.948
	Outpatient	8228.45	6386.13	4844.57	
Treatment category	Traditional Chinese Medicine	11341.95	9277.12	7234.55	0.023
	Integrated TCM and Western medicine	8189.68	6358.10	4769.66	
Discharge method	Discharge by doctor’s advice	8275.64	6283.01	4920.18	0.070
	Transfer by doctor’s advice	7570.61	7433.01	3002.15	
Application of TCM characteristic techniques	Yes	8153.15	6279.64	4478.47	<0.001
	No	9560.27	12044.80	8429.78	
TCM hospital preparations	Yes	8537.09	6754.90	4476.92	<0.001
	No	6826.96	4588.20	5183.15	
TCM diagnostic and treatment equipment	Yes	7780.78	6283.01	4453.57	0.158
	No	9385.30	6730.82	6686.15	

### 3.4 Distribution of the dependent variable and model fit evaluation for Xiangbi

The minimum total hospitalization cost was 316.33 yuan, the maximum was 60591.01 yuan, the mean was 8237.90 yuan, and the standard deviation was 7277.18 yuan. The data exhibited a markedly right-skewed distribution with substantial variability, indicating the appropriateness of adopting a generalized linear model with a Gamma distribution. The goodness-of-fit evaluation results showed that the deviance/degrees of freedom ratio was 0.327 and the Pearson chi-square/degrees of freedom ratio was 0.490; both ratios were less than 1, indicating good overall model fit without significant overdispersion, as shown in Tables 4, 5.

**Table 4:** Descriptive statistics of the dependent variable.

Variable	*N*	Minimum (RMB)	Maximum (RMB)	Mean (RMB)	Standard deviation
Total hospitalization cost (RMB)	1,046	316.33	60591.01	8237.8977	7277.17844

**Table 5:** Model goodness of fit.

Indicator	Value	Degrees of freedom	Value/df
Deviance	334.873	1,025	0.327
Scaled deviance	683.480	1,025	–
Pearson chi-square	502.202	1,025	0.490
Scaled Pearson chi-square	1025.000	1,025	–

### 3.5 Omnibus test

The Omnibus test showed that the likelihood ratio Chi-square value was 298.113, with 20 degrees of freedom, *p* < 0.001, indicating that the independent variables included in the model collectively had a significant explanatory effect on hospitalization costs, as shown in Table 6.

**Table 6:** Omnibus test.

Test statistic	Value	Degrees of freedom	Degrees of freedom
Likelihood ratio chi-square	298.113	20	<0.001

### 3.6 Independent effects on hospitalization costs

Type III Wald test results showed that, after controlling for other variables, length of hospital stay (Wald χ^2^ = 117.860, *p* < 0.001), year (Wald χ^2^ = 10.859, *p* = 0.004), age group (Wald χ^2^ = 23.799, *p* = 0.001), application of TCM characteristic techniques (Wald χ^2^ = 7.240, *p* = 0.007), use of hospital-prepared Chinese medicine preparations (Wald χ^2^ = 9.302, *p* = 0.002), and application of TCM diagnosis and treatment equipment (Wald χ^2^ = 7.539, *p* = 0.006) were independent influencing factors of hospitalization costs. In contrast, the number of hospitalizations, sex, marital status, admission route, treatment category, discharge method, and payment method of medical expenses did not have significant independent effects on costs (*p* > 0.05). The specific parameter estimates are presented in Tables 6, 7. Some variables showed inconsistent results between the univariate and multivariate analyses. Marital status was significant in the univariate analysis (*p* < 0.001) but became non-significant after controlling for other variables (*p* = 0.355). One possible explanation—though speculative and not formally tested in this study—is that the effect of marital status on costs may be mediated or confounded by factors such as age and length of stay, as patients with spouses tended to be older and have longer hospital stays. The application of TCM diagnosis and treatment equipment was not significant in the univariate analysis (*p* = 0.158) but was significant in the multivariate analysis (*p* = 0.006). A speculative hypothesis for this discrepancy is that the application of TCM diagnosis and treatment equipment may have negative interactions with other variables (e.g., application of TCM characteristic techniques and use of hospital-prepared Chinese medicine preparations)—that is, patients using such equipment often have more complex conditions and receive more comprehensive TCM treatments, and these factors may have offset the independent effect of the equipment in the univariate analysis. After controlling for these confounders in the multivariate model, the independent effect of the equipment emerged. However, these interpretations remain speculative hypotheses rather than formally validated findings, as no interaction tests or mediation analyses were conducted in the current study (Table 8).

**Table 7:** Type III Wald test.

Source of effect	Wald Chi-Square	df	*p*-value
(Intercept)	3711.536	1	<0.001
Sex	2.719	1	0.099
Age	23.799	6	0.001
Marital status	0.854	1	0.355
Length of stay	117.860	1	<0.001
Number of admissions	1.058	1	0.304
Payment method for medical expenses	3.170	2	0.205
Year	10.859	2	0.004
Admission route	0.000	1	0.989
Treatment category	2.455	1	0.117
Discharge method	0.230	1	0.631
Use of TCM characteristic techniques (yes/no)	7.240	1	0.007
Use of hospital-prepared TCM preparations (yes/no)	9.302	1	0.002
Use of TCM diagnostic and therapeutic equipment (yes/no)	7.539	1	0.006

**Table 8:** Parameter estimates.

Parameter		*B*	Std. error	Significance	ExP(B)	95% CI lower	95% CI upper
(Intercept)		9.788	0.3993	<0.001	17825.646	9.006	10.571
Sex (ref: female)	Male	0.074	0.0449	0.099	1.077	−0.014	0.162
Age (ref: ≥81)	≤30	−0.301	0.3736	0.420	0.740	−1.033	0.431
	31–40	−0.028	0.3670	0.939	0.972	−0.748	0.691
	41–50	0.040	0.3665	0.912	1.041	−0.678	0.759
	51–60	0.081	0.3665	0.826	1.084	−0.638	0.799
	61–70	0.152	0.3680	0.680	1.164	−0.569	0.873
	71–80	0.142	0.3726	0.703	1.153	−0.588	0.872
Marital status (ref: without spouse)	With spouse	0.068	0.0735	0.355	1.070	−0.076	0.212
Length of stay (ref: ≥14 days)	1 ~ 13 days	−0.936	0.0862	<0.001	0.392	−1.105	−0.767
Number of admissions (ref: ≥2)	1 admission	−0.082	0.0800	0.304	0.921	−0.239	0.075
Payment method (ref: New Rural Cooperative Medical Scheme)	Urban resident basic medical insurance	0.159	0.1112	0.153	1.172	−0.059	0.377
	Urban employee basic medical insurance	0.191	0.1118	0.087	1.211	−0.028	0.410
Year (ref: 2025)	2023	0.304	0.0930	0.001	1.355	0.122	0.486
	2024	0.015	0.0517	0.775	1.015	−0.087	0.116
Admission route (ref: outpatient)	Emergency	−0.003	0.1982	0.989	0.997	−0.391	0.386
Treatment category (ref: integrated Chinese and Western medicine)	TCM	0.280	0.1788	0.117	1.323	−0.070	0.631
Discharge method (ref: referred by medical order)	Discharged by medical order	−0.047	0.0984	0.631	0.954	−0.240	0.146
Use of TCM characteristic techniques (ref: no)	Yes	−0.257	0.0955	0.007	0.773	−0.444	−0.070
Use of hospital-prepared TCM preparations (ref: no)	Yes	0.184	0.0604	0.002	1.202	0.066	0.302
Use of TCM diagnostic and therapeutic equipment (ref: no)	Yes	−0.146	0.0533	0.006	0.864	−0.251	−0.042

B represents the unstandardized regression coefficient estimated from the generalized linear model with Gamma distribution and log link function. Standard errors and 95% confidence intervals are reported. Exp(B) represents the multiplicative effect on the predicted cost for a one-unit change in the predictor.

## 4 Discussion

### 4.1 Leveraging the advantages of TCM characteristic techniques, optimizing the cost structure, and continuously reducing the proportion of drug and consumable expenses

The results showed that the application rate of TCM characteristic techniques among patients with cervical spondylosis was as high as 94.0%, and the proportion of TCM treatment costs in hospitalization expenses had risen to 32.46%. However, Western medicine and consumable costs still accounted for a certain proportion, indicating there is still room for optimization of the cost structure. In this regard, the following recommendations are proposed: first, implement centralized procurement policies for drugs and consumables while addressing the needs of medical staff, improve the hospital compensation mechanism, and further reduce drug and consumable expenses (9); second, deepen the zero-markup policy for drugs and consumables, raise the prices of medical services that reflect labor value, strengthen government compensation, and reinforce cost control in public hospitals (10); third, promote volume-based procurement of drugs and consumables, use information technology to monitor cost composition, guide cost control through performance incentives, and assess the proportion of drug and consumable expenses (11); fourth, pay attention to the driving effect of consumable costs on total expenses, strengthen refined management of consumables, and standardize treatment procedures (12); fifth, take into account the cost advantages of TCM characteristic therapies, avoid simplistic control of the drug cost proportion, and reasonably adjust the prices of TCM medical services (13).

### 4.2 Taking length of stay as the core control target and promoting refined management of TCM clinical pathways

Gamma generalized linear model analysis indicated that length of stay was the most significant independent factor influencing hospitalization costs for cervical spondylosis (Wald χ^2^ = 117.860, *p* < 0.001), which is highly consistent with existing literature. Focusing on this core point, it is recommended to strengthen clinical pathway management, reduce ineffective hospital days, and increase bed turnover rate (14); optimize and improve the clinical pathways for TCM dominant diseases and align them with the reform of DIP/DRG payment methods (15); promote pre-hospitalization models and day surgery to optimize medical service processes and shorten length of stay (16); implement scheduled examinations and strengthen bidirectional referral to reasonably reduce hospital days (17); meanwhile, for long-stay patients, consider per-diem disease-based payment and establish a dynamic management mechanism for TCM dominant diseases (15).

### 4.3 Establishing differentiated evaluation and tiered payment mechanisms to fully account for cost heterogeneity related to age

The results showed that age group was a significant independent factor influencing hospitalization costs for cervical spondylosis (Wald χ^2^ = 23.799, *p* = 0.001), with significant cost differences among different age groups. Accordingly, it is recommended to establish differentiated evaluation indicators and implement tiered management for hospitals of different levels and patients of different age groups (18); pay attention to differences among hospitals at various levels, implement differentiated reforms, expand the scope of TCM dominant diseases in tertiary hospitals, and raise the fixed payment standards (19); strengthen health security for the older adults, and provide reasonable medical insurance payment preferences and flexible management for older patients (20); incorporate demographic characteristics such as age and sex into DIP/DRG grouping factors to more accurately reflect differences in resource consumption (21); dynamically adjust disease-based payment standards and continuously monitor the long-term outcomes of the reform (18).

### 4.4 Improving supportive policies for the application of TCM characteristic techniques and establishing an incentive mechanism for the labor value of TCM techniques

The application of TCM characteristic techniques, the use of TCM preparations in medical institutions, and the use of TCM diagnosis and treatment equipment were all significant independent factors influencing hospitalization costs (all *p* < 0.01). Moreover, patients who did not receive TCM characteristic techniques incurred even higher costs, suggesting the potential advantage of TCM techniques in cost control. It is feasible to establish differentiated payment standards and dynamic adjustment mechanisms for TCM dominant diseases under the DRG framework to fully reflect the labor value of TCM characteristic therapies (22); improve the dynamic adjustment mechanism for the prices of TCM treatment services to reflect the labor value of medical staff (23); appropriately raise the add-on coefficient for TCM medical institutions to reflect medical insurance preferences for traditional Chinese medicine (23); establish a TCM efficacy-based payment mechanism grounded in scientific pricing, link payment standards to actual medical costs, and appropriately set “efficiency and price increase” payment standards (24); and build a multi-party negotiation mechanism through which medical insurance departments and TCM medical institutions can negotiate to reasonably determine the payment standards for TCM dominant diseases, with consideration given to establishing a separate fund pool (13).

## 5 Limitations and prospects

### 5.1 Study limitations

(1) Limited regional and institutional representativeness of the data. The data in this study were derived from a tertiary Grade A Traditional Chinese Medicine (TCM) hospital in Z Province. As an economically developed province, Z Province may differ from other regions in China in terms of DRG implementation progress and medical insurance payment standards. Moreover, significant differences exist between tertiary TCM hospitals and primary-level TCM hospitals in disease admission structure, diagnosis and treatment pathways, and cost composition. Therefore, caution should be exercised when generalizing the conclusions of this study to other regions or primary healthcare institutions.
(2) Lack of key clinical and efficacy indicators. Limited by the availability of data from the medical record front page and medical insurance claims, this study focused on hospitalization costs and their structure, and did not include clinical and service quality indicators such as disease severity, comorbidity index, functional recovery, patient satisfaction, and readmission rate. There is an inherent tension between cost containment and the assurance of therapeutic efficacy. The absence of an efficacy dimension analysis may affect the overall judgment of the reasonableness of cost control. This study did not include any clinical outcome measures; therefore, it cannot assess net benefits or cost-effectiveness of the observed cost changes.
(3) Insufficient capacity for causal inference. Although the generalized linear model identified independent associations between hospitalization costs and factors such as length of stay, year, age, and application of TCM techniques, it could not fully eliminate the interference of confounding factors. More robust study designs are needed to verify the findings.
(4) No formal interaction or mediation analyses were conducted in this study; therefore, the relationships between variables should be interpreted with caution.

### 5.2 Research prospects

(1) Promote multi-center, multi-level healthcare institution research designs. Future studies can expand the scope to multiple regions within the province and even nationwide, covering medical institutions of different levels and types, to build a more representative sample. This will provide a more solid data foundation for formulating region-specific and tiered DRG payment standards and management strategies for TCM dominant diseases.
(2) Extend the study time span to achieve long-term dynamic evaluation of policy effects. Continuously track the cost structure, service volume, and quality indicators of cervical spondylosis and other TCM dominant diseases over an extended period. It is advisable to incorporate quasi-experimental methods such as interrupted time series analysis and difference-in-differences to more clearly identify the net effects at each stage of policy implementation and provide a basis for dynamically adjusting payment standards.
(3) Integrate clinical data and cost data to construct a bidirectional “cost–efficacy” evaluation framework. On the basis of medical insurance claims and medical record front page data, establish linkages with electronic medical records, specialty scale assessments, and follow-up systems, and introduce clinical dimension indicators. While controlling costs, ensure that the clinical efficacy of TCM is objectively measured, thereby contributing to the construction of a “value-based” TCM payment model.
(4) Explore the formation of management strategies and policy plans for disease-based payment for TCM dominant diseases. Based on key cost-influencing factors such as length of stay, age, and the application of TCM techniques, attempt to construct payment models with different risk stratifications. Promote the design of supporting mechanisms such as hierarchical diagnosis and treatment, the linkage between outpatient and inpatient services, pre-hospitalization, and day wards, ultimately establishing a replicable and scalable implementation plan for DRG payment reform for TCM dominant diseases.

## Data Availability

The data analyzed in this study are not publicly available due to patient privacy and institutional data protection policies.
